# Xerogel-Sequestered Silanated Organochalcogenide Catalysts for Bromination with Hydrogen Peroxide and Sodium Bromide

**DOI:** 10.3390/molecules20069616

**Published:** 2015-05-26

**Authors:** Caitlyn M. Gatley, Lisa M. Muller, Meredith A. Lang, Eduardo E. Alberto, Michael R. Detty

**Affiliations:** Department of Chemistry, The State University of New York at Buffalo, Buffalo, NY 14260, USA; E-Mails: cgatley@buffalo.edu (C.M.G.); lmmuller@buffalo.edu (L.M.M.); malang@buffalo.edu (M.A.L.); albertoee@gmail.com (E.E.A.)

**Keywords:** organoselenides, organotellurides, diorgano diselenides, xerogel, catalytic bromination

## Abstract

While H_2_O_2_ is a powerful oxidant, decomposing into environmentally benign H_2_O and O_2_, a catalyst is often required for reactions with H_2_O_2_ to proceed at synthetically useful rates. Organotellurium and organoselenium compounds catalyze the oxidation of halide salts to hypohalous acids using H_2_O_2_. When sequestered into xerogel monoliths, the xerogel-chalcogenide combinations have demonstrated increased catalytic activity relative to the organochalcogen compound alone in solution for the oxidation of halide salts to hypohalous acids with H_2_O_2_. Diorganotellurides, diorganoselenides, and diorganodiselenides bearing triethoxysilane functionalities were sequestered into xerogel monoliths and their catalytic activity and longevity were investigated. The longevity of the catalyst-xerogel combinations was examined by isolating and recycling the catalyst-xerogel combination. It was found tellurium-containing catalyst **3** and selenium-containing catalyst **8** maintained their catalytic activity through three recycling trials and adding electron-donating substituents to catalyst **3** also increased the catalytic rate. The presence of organotellurium and organoselenium groups in the +4 oxidation state was determined by X-ray photoelectron spectroscopy.

## 1. Introduction

The use of H_2_O_2_ as an oxidant relative to other oxidizing agents has attracted a great deal of attention due to the advantages of using the environmentally benign H_2_O_2_, which decomposes into water and oxygen [1]. While H_2_O_2_ is a powerful oxidant thermodynamically, kinetically, H_2_O_2_ oxidations can be slow and often require a catalyst to accelerate the reactions to synthetically useful rates. Selenium-containing organic compounds have been used as catalysts for the activation of H_2_O_2_ for Baeyer-Villiger oxidations [2], epoxidation reactions [3], conversion of sulfides to sulfoxides [4] and sulfones [5], aldehydes to carboxylic acids [6], and secondary amines to nitrones [7]. The selenium in glutathione peroxidase is responsible for protecting cell membranes from oxidative damage by reducing a variety of hydroperoxides and in turn oxidizing thiols to disulfides. A variety of organoselenium mimics of glutathione peroxidase have been shown to produce the same activity [8,9,10].

Selenoxides [11,12,13] arylseleninic acids [14,15], and diorganotellurides [16,17,18] are also effective catalysts with H_2_O_2_ for the oxidation of halide salts. These reactions proceed at ambient conditions to form hypohalous acid, which can be used as a halogenating agent. This procedure offers a less hazardous, more environmentally friendly alternative to the use of other halogenating agents such as elemental bromine. One selenoxide was shown to have a greater catalytic effect when sequestered in a xerogel—an interconnected matrix created from the formation of siloxane bonds [19]. The selenoxide was incorporated into the gel matrix utilizing an alcohol functionality, providing one potential covalent bond to hold the catalyst to the structure. The xerogel provides a porous material that can create local environments of higher reaction concentrations than bulk solutions, which may increase the rate of oxidation of halide salts with H_2_O_2_.

Xerogel coatings are effective fouling-release materials for use in marine environments [20,21,22,23,24,25,26,27]. The incorporation of a telluride or selenoxide catalyst into the xerogel has been shown to provide statistically significant anti-fouling character to the material as the telluride or selenoxide can react with ambient H_2_O_2_ and halides, naturally occurring in sea water, to produce hypohalous acids [28]. The resulting oxidizing surface may provide negative settlement cues to marine organisms. For chalcogen-containing compounds to be effective catalysts in the marine environment, they need not only to produce functional concentrations of hypohalous acids but also remain strongly bound to the xerogel for coating longevity and to avoid contamination of the local marine environment with chalcogen-containing compounds.

In this study, we compare the catalytic activity of tellurides, selenoxides, and seleninic acids for the oxidation of NaBr with H_2_O_2_ when covalently incorporated into a xerogel matrix via a trialkoxysilane group. The longevity of the catalyst-xerogel combinations was examined by isolating and recycling the catalyst-xerogel combination. The presence of organotellurium and organoselenium groups in the +4 oxidation state was determined by X-ray photoelectron spectroscopy.

## 2. Results and Discussion

### 2.1. Synthesis of Chalcogenide Catalysts and Catalyst Precursors

**Figure 1:**
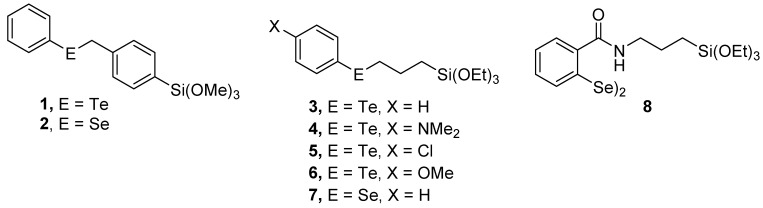
Organochalcogenides used as catalysts or catalyst precursors in xerogel matrices.

The active catalysts in the xerogel monoliths are the telluroxide (prepared from the corresponding telluride) [12,16,17] the selenoxide (prepared via oxidation of the corresponding selenide) [11,12,13,18] and the seleninic acid (prepared from oxidation of the corresponding diselenide) functional groups [14,15]. We envisioned that the active catalysts or catalyst precursors would arise from phenyl benzyl telluride **1**, phenyl benzyl selenide **2**, aryltellurides **3**–**6**, phenylselenide **7**, and diselenide **8** (Figure 1). All of these compounds bear an alkyl(triethoxy)silyl or alkyl(trimethoxy)silyl group, which can form sols by oligomerization. Our attempts to isolate these compounds following synthesis were unsuccessful. While the desired compound could be detected by mass spectrometry in several cases, siloxane hydrolysis was rapid, as was sol formation. As a consequence, the materials were generated *in situ* and incorporated directly into the xerogels.

**Scheme 1:**
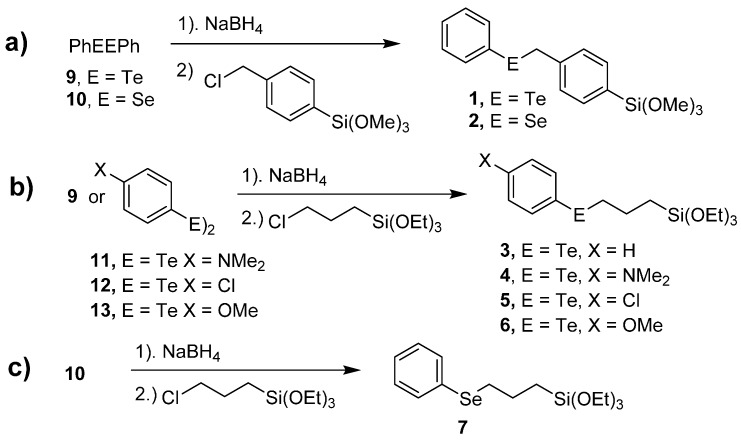
Synthesis of (**a**) benzyl phenyl catalysts or catalyst precursors **1** and **2**; (**b**) telluride catalysts **3**–**6**; and (**c**) selenide catalyst precursor **7**.

The phenyl benzyl chalcogenides **1** and **2** were prepared as shown in Scheme 1a. Reduction of diphenyl ditelluride (**9**) or diphenyl diselenide (**10**) with sodium borohydride gave the corresponding sodium phenylchalcogenide, which was added to (*p*-chloromethyl)phenyltrimethoxysilane to give telluride **1** or selenide **2**. Similarly, reduction of ditelluride **9** or diaryl ditellurides **11**–**13** with sodium borohydride and addition of the resulting sodium aryl chalcogenide to 3-chloropropyl(triethoxy)silane gave telluride catalysts **3**–**6** (Scheme 1b). Reduction of diselenide **10** with sodium borohydride and addition of the resulting sodium phenylselenide to 3-chloropropyl(triethoxy)silane gave selenide **7** (Scheme 1c).

**Scheme 2:**
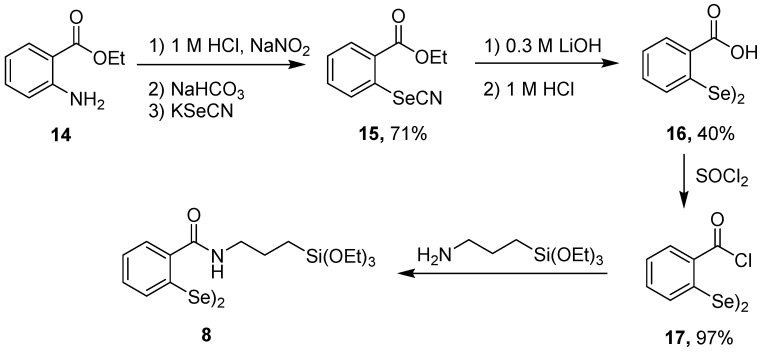
Synthesis of diselenide **8**.

The synthesis of diselenide **8** followed a different route as shown in Scheme 2. Diazotization of ethyl 2-aminobenzoate (**14**) followed by treatment with KSeCN gave selenocyanate **15** in 71% isolated yield. Hydrolysis of **15** with 0.3 M LiOH, followed by treatment with 1 M HCl gave di-2-carboxyphenyl diselenide (**16**) in 40% isolated yield. The carboxylic acid functionality was converted to the diacid chloride **17** with thionyl chloride in 97% isolated yield. The addition of **17** to 3-aminopropyl(triethoxy)silane in dichloromethane gave **8**.

### 2.2. Preparation of Xerogel Monoliths with Chalcogenide Catalysts/Catalyst Precursors

The catalysts/catalyst precursors were incorporated directly into a tetraethoxysilane (TEOS) xerogel. For **1** and **3**–**6**, 0.5 mmol of aryltelluride in its original reaction mixture and 20 mmol of TEOS were treated with ammonium hydroxide for 24 h and the resulting sol was placed in an 8 dram (1 fl. oz or 3.7 mL) vial. Solvents were allowed to evaporate for 10 days and further removal of solvents was performed under vacuum at ambient temperature for 48 h. The resulting monoliths were crushed with a mortar and pestle to give a fine white powder. For **2** and **7**, 0.2 mmol of selenide in its original reaction mixture and 40 mmol of TEOS were treated with ammonium hydroxide for 24 h to prepare the sol, which was placed in an 8 dram vial. Solvents were allowed to evaporate for 10 days and further removal of solvents was performed under vacuum at ambient temperature for 48 h and, again, monoliths were crushed with mortar and pestle to give a fine white powder. For diselenide **8**, 0.2 mmol of **8** in its original reaction mixture and 40 mmol of TEOS were treated with aqueous HCl for 24 h to prepare the sol, which was then treated as described above. The final concentration of catalyst/catalyst precursor was 2.5 mol % relative to TEOS for tellurides **1** and **3**–**6** and 0.5 mol % relative to TEOS for selenides **2** and **7** and diselenide **8**. 

### 2.3. Catalytic Oxidation of Bromide with Hydrogen Peroxide

**Scheme 3:**
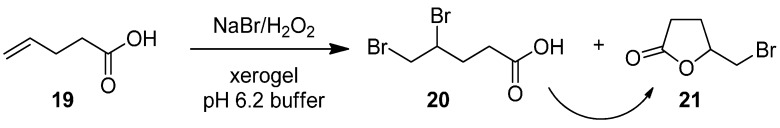
Bromination of 4-pentenoic acid (**19**) as an indirect measure of bromine/hypobromous acid production from the oxidation of bromide with hydrogen peroxide.

The ability of xerogel-sequestered catalysts **1**–**8** to catalyze the oxidation of bromide to positive bromine species with H_2_O_2_ was evaluated indirectly via the bromination of 4-pentenoic acid (**19**) to give a mixture of **20** and **21** (Scheme 3). This indirect method has been used by us in prior work [11,12,13,14,15,16,17,18] and allows results to be compared directly for the many different catalyst systems. The bromination of **19** at 25 ± 1 °C as shown in Scheme 3 was followed using ^1^H-NMR spectroscopy. The initial mixture of 4,5-dibromopentanoic acid (**20**) and bromolactone **21** was converted to **21** as the sole product under the reaction conditions described here. Propionic acid (0.01 M), which was unreactive under these conditions, was added as an internal standard for the integration of ^1^H-NMR signals. Results are compiled in Table 1 for TEOS xerogels incorporating catalysts/catalyst precursors **1**–**8**.

**Table 1:** Pseudo first-order rate constants (*k*_obs_) and relative rates (*k*_rel_) for bromination of 4-pentenoic acid (**19**) with TEOS xerogel-sequestered catalysts/catalyst precursors **1**–**8** compared to a catalyst-free TEOS xerogel control in pH 6.2 phosphate buffer ^a^.

Catalyst	*k*_obs_ (s^−1^) ^c^	*k*_rel_
**blank**	(8.35 ± 0.17) × 10^−7^	1.0
**1 ^a^**	(5.71 ± 0.02) × 10^−5^	68
**2 ^b^**	(1.27 ± 0.20) × 10^−5^	15
**3 ^a^**	(1.91 ± 0.06) × 10^−5^	23
**4 ^a^**	(3.64 ± 0.54) × 10^−5^	44
**5 ^a^**	(3.61 ± 0.01) × 10^−6^	4.3
**6 ^a^**	(6.93 ± 0.20) × 10^−6^	8.3
**7 ^b^**	(8.03 ± 1.11) × 10^−6^	9.6
**8 ^b^**	(1.37 ± 0.02) × 10^−5^	16

^a^ Catalyst (0.02 eq relative to substrate), 4-pentenoic acid (**19**, 0.14 M), NaBr (1.4 M), H_2_O_2_ (0.35 M); ^b^ Catalyst (0.025 eq relative to substrate), 4-pentenoic acid (**19**, 0.14 M), NaBr (1.4 M), H_2_O_2_ (0.35 M); ^c^ *k*_obs_ is the average of duplicate runs, T = 25 ± 1 °C.

The results in Table 1 show that the **TEOS**-**1**–**8** combinations are all catalytically active and accelerate the rate of bromination of **19** relative to the catalyst-free, TEOS blank. In comparing the phenyl benzyl telluride and selenide derivatives **1** and **2**, respectively, the **TEOS-1** xerogel catalyst accelerates bromination more than any of the other xerogel catalysts and gives a 4.5-fold greater rate enhancement than the xerogel containing selenide **2**. Both catalysts are presumably oxidized to the telluroixde/selenoxide derivatives to give the active catalyst. In comparing the phenyl propyl telluride and selenide derivatives **3** and **7**, respectively, the telluride **TEOS**-**3** combination gives a 2.4-fold rate enhancement relative to the selenide **TEOS**-**7** combination. Again, the telluride and selenide functionality in **3** and **7** are presumably oxidized to the telluroxide and selenoxide, respectively. A comparison of the **TEOS**-**1** and **TEOS**-**3** combinations shows a 3-fold faster rate for the benzyl derivative **1**. Similarly, a comparison of the **TEOS**-**2** and **TEOS**-**7** combinations shows a 1.4-fold faster rate for **2**. Based on these comparisons, the telluride-containing xerogels appears to be better catalysts than the corresponding selenide-containing xerogels and the benzyl group appears to be better for catalytic activity than a propyl group.

Within the telluride **TEOS**-**3**–**6** series, substituents on the aryl group impact the rates of bromination shown in Table 1. The electron-donating 4-*N*,*N*-dimethylamino substituent in **4** gives a two-fold rate acceleration relative to the unsubstituted **3** and a ten-fold acceleration relative to the electron-withdrawing chloro substituent in **5**.

The diselenide **8** would give two equivalents of seleninic acid upon oxidation with peroxide and the seleninic acid would be the functional catalyst in the system. Consequently, the xerogel monoliths were added to give 2.5 mol % of **2** or **7** relative to **19** and 1.25 mol% of **8** (2.5 mol % of the seleninic acid formed *in situ*). The **TEOS-2** and **TEOS-8** xerogels had very similar catalytic activity as indicated by the rates of bromination in Table 1, while the xerogel containing **7** was only about 65% as active as the xerogels containing **2** or **8**.

### 2.4. Catalytic Lifetimes and Recyclability

Another comparison to make among the xerogel-sequestered telluroxide, selenoxide, and seleninic acid catalysts is the ability to recover and recycle the catalyst. The ideal catalyst would remain sequestered in the xerogel and would not undergo chemical reactions to give new functionalities. Recovered and recycled catalyst should give essentially identical rates of bromination as if the xerogel-sequestered catalyst were “original.” The originally made xerogel-sequestered catalysts **1**–**3**, **7**, and **8** were recovered and recycled an additional three times using the same concentration of reagents. Values of *k*_obs_ for each recycle and *k*_rel_ relative to the initial reaction are compiled in Table 2. The recyclability of catalysts **4**–**6** were not evaluated as the stability of the propyl linker connecting the tellurium to the silane functionality was already evaluated using the “parent” telluride catalyst **3**.

The xerogel-sequestered phenyl benzyl chalcogenides **1** and **2** did not recycle well. The first recycling showed 20% of the initial reactivity for the **TEOS**-**1** combination and 27% of the initial reactivity for the **TEOS-2** combination. The second and third recycling of these materials gave further decreases in reactivity. The phenyl propyl selenide in the **TEOS-7** combination behaved similarly. The first recycle showed 55% of the initial rate and, in two subsequent recycles, the rate decreased to 45% of the initial rate. In contrast, the phenyl propyl telluride in the **TEOS-3** combination and the seleninic acid from the **TEOS-8** combination gave similar rates for the initial reaction and for each of the three recycles (Table 2).

**Table 2:** Observed pseudo first-order rate constants (*k*_rel_) and relative rates (*k*_rel_) for bromination of 4-pentenoic acid (**19**) with TEOS xerogel-sequestered **1**–**3**, **7**, and **8** for initial reaction and three recycles of the xerogel-catalyst combination in pH 6.2 phosphate buffer ^a^.

Cycle	Catalyst	*k*_obs_ (s^−1^) ^d^	*k*_rel_
Initial Reaction ^a^	**1**	(5.71 ± 0.02) × 10^−5^	1.00
First Recycle ^a^	**1**	(1.16 ± 0.01) × 10^−5^	0.20
Second Recycle ^a^	**1**	(1.08 ± 0.20) × 10^−5^	0.19
Third Recycle ^a^	**1**	(5.69 ± 0.50) × 10^−6^	0.10
Initial Reaction ^b^	**2**	(1.27 ± 0.20) × 10^−5^	1.00
First Recycle ^b^	**2**	(3.44 ± 0.05) × 10^−6^	0.27
Second Recycle ^b^	**2**	(2.53 ± 0.01) × 10^−6^	0.20
Third Recycle ^b^	**2**	(1.96 ± 0.05) × 10^−6^	0.15
Initial Reaction ^a^	**3**	(1.91 ± 0.06) × 10^−5^	1.00
First Recycle ^a^	**3**	(2.48 ± 0.06) × 10^−5^	1.30
Second Recycle ^a^	**3**	(1.68 ± 0.09) × 10^−5^	0.88
Third Recycle ^a^	**3**	(2.00 ± 0.03) × 10^−5^	1.05
Initial Reaction ^b^	**7**	(8.03 ± 0.55) × 10^−6^	1.00
First Recycle ^b^	**7**	(4.45 ± 0.05) × 10^−6^	0.55
Second Recycle ^b^	**7**	(3.65 ± 0.44) × 10^−6^	0.45
Third Recycle ^b^	**7**	(3.65 ± 0.41) × 10^−6^	0.45
Initial Reaction ^c^	**8**	(1.24 ± 0.01) × 10^−5^	1.00
First Recycle ^c^	**8**	(1.34 ± 0.00) × 10^−5^	1.08
Second Recycle ^c^	**8**	(1.14 ± 0.00) × 10^−5^	0.92
Third Recycle ^c^	**8**	(1.46 ± 0.02) × 10^−5^	1.18

^a^ catalyst (0.02 eq relative to substrate), 4-pentenoic acid (**19**, 0.14 M), NaBr (1.4 M), H_2_O_2_ (0.35 M); ^b^ catalyst (0.025 eq relative to substrate), 4-pentenoic acid (**19**, 0.14 M), NaBr (1.4 M), H_2_O_2_ (0.35 M); ^c^ catalyst (0.025 eq relative to substrate), 4-pentenoic acid (**19**, 0.14 M), NaBr (2.1 M), H_2_O_2_ (0.21 M); ^d^ *k*_obs_ is the average of duplicate runs, T = 25 ± 1 °C.

**Scheme 4:**
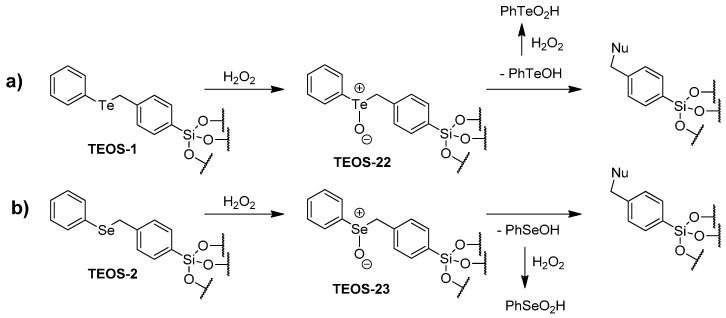
Potential routes to loss of catalytic activity (**a**) with phenyl benzyl telluride catalyst **TEOS-1** or (**b**) phenyl benzyl selenide catalyst **TEOS-2**.

The phenyl benzyl chalcogenides **1** and **2** did not recycle well when sequestered in a TEOS xerogel. Oxidation to the telluroxide **TEOS-22** of **TEOS-1** or the selenoxide **TEOS-23** of **TEOS-2** would create a situation where nucleophilic substitution (perhaps with bromide) at the benzylic position would release phenyltellurenic acid (PhTeOH) from **TEOS-22** or phenylselenenic acid (PhSeOH) from **TEOS-23** and leave a non-catalytic site in the TEOS xerogel as illustrated in Scheme 4. Both PhTeOH and PhSeOH could function as catalysts (or be oxidized to tellurinic acid or seleninic acid oxidation state), which would contribute to the initial rates of reaction. Recovery and recycling would eliminate the non-sequestered catalyst and the subsequent reaction would have less chalcogenide to catalyze the bromination. The most accessible nucleophilic sites would be lost first and it is not surprising that rate of loss of catalytic activity slows with subsequent recyclings.

While we were unable to detect PhTeOH, PhTeO_2_H, PhSeOH, or PhSeO_2_H following exposure of **TEOS-1** or **TEOS-2** to H_2_O_2_, we were able to demonstrate the facile cleavage of the Te(IV)-benzyl carbon bond. Phenylbenzyltelluride and phenylbenzylselenide are oxidized to PhTeCl_2_CH_2_Ph and PhSeCl_2_CH_2_Ph, respectively, with chlorine. Heating these chalcogen(IV) compounds leads to formation of PhTeCl or PhSeCl and ClCH_2_Ph, which is analogous to the chemistry proposed for the degradation of **TEOS-22** and **TEOS-23**.

**Scheme 5:**
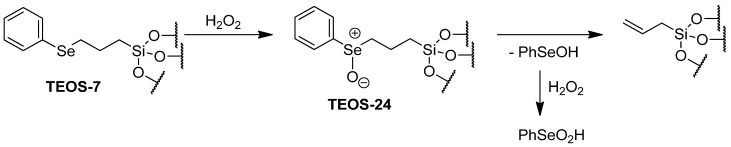
Potential routes to loss of catalytic activity with phenyl propyl selenide in **TEOS-7**.

The phenyl propyl chalcogenide catalysts based on **3** and **7** behave quite differently from one another. Oxidation of the selenide in **TEOS-7** to the selenoxide in **TEOS-24** gives the active catalyst. The first recycling trial for **TEOS-7** demonstrated a 45% reduction in reaction rate. The loss in catalytic activity is most likely due to the loss of the selenide functionality in the **TEOS-7** xerogel matrix through *syn*-elimination of PhSeOH [29,30,31,32] to produce an alkene as shown in Scheme 5. Oxidation of PhSeOH to the seleninic acid PhSeO_2_H would give a catalyst that would be present in solution during the initial reaction, but would be removed during the recovery and recycling of the xerogel-catalyst combination. In the initial reaction, all the selenoxide functionality properly aligned for *syn*-elimination would be first to react. Subsequent *syn*-elimination from selenoxides in further recyclings would be from those less favorably aligned. Telluroxides do not undergo *syn*-elimination reactions as easily as selenoxides do. The **TEOS**-**3** as a consequence keeps the telluride sequestered and the catalytic activity of the **TEOS**-**3** combination remains constant through three recyclings of the catalyst with complete consumption of **19**.

**Scheme 6:**
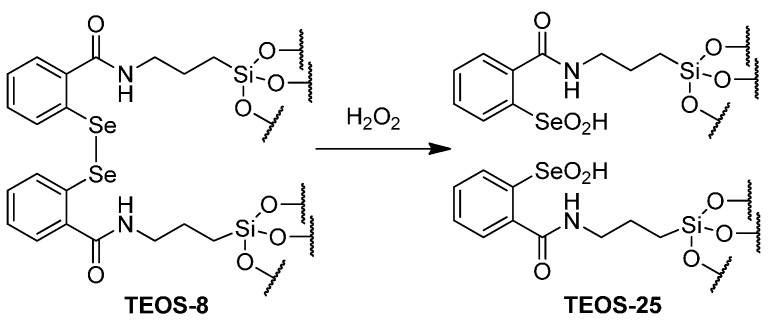
Oxidation of **TEOS-8** with H_2_O_2_ to seleninic-acid containing **TEOS-25**.

Oxidation of diselenide xerogel **TEOS-8** by hydrogen peroxide would generate two equivalents of xerogel-sequestered seleninic acid as shown in Scheme 6 for the generation of **TEOS-25**. The seleninic acids do not undergo *syn*-elimination and the seleninic acid remains sequestered in the TEOS. Consequently, the rates of bromination are essentially unchanged from the initial reaction through three recyclings.

### 2.5. Oxidation-State Changes as Measured by X-ray Photoelectron Spectroscopy in the TEOS Xerogel/Chalcogenide Catalysts upon Exposure to Hydrogen Peroxide

Since the oxidation of the telluride to telluroxide [12,15,16,17] selenide to selenoxide [11,12,13] and diselenide to seleninic acid [14,15] are essential steps in catalytic cycle for the formation of the hypohalous acid, it was of interest to monitor the formation of the Se(IV) and Te(IV) oxidation states utilizing X-ray photoelectron spectroscopy (XPS). XPS spectra of 20 mol % **7** in TEOS and 5 mol % **3** in TEOS and coated on glass slides were recorded. Spectra were also taken of the same coatings after soaking in 1.0 × 10^−4^ M H_2_O_2_ for 24 h. The ratios of the Te(II) to Te(IV) and Se(II) to Se(IV) before and after soaking are listed in Table 3 and Table 4, respectively. The alkoxysilanes polymerize and incorporate the catalyst randomly into the coating. Therefore, the catalyst is randomly distributed in three dimensions. Since XPS has the capacity to detect atoms to a depth of ~10 nm, [33] spectra show atoms both at the surface and those below the surface that are less prone to interacting with the surrounding chemical environment—*i.e.*, are less prone to oxidation. 

**Table 3:** The position and ratios of Te(II) and Te(IV) calculated for samples “as prepared” and after submersion in 1.0 × 10^−4^ M H_2_O_2_ for 24 h. The position and ratio values are the mean of five spectra recorded for each sample ± 1 SD. The peak positions were referenced by setting a C 1s peak to 284.5 eV.

“as Prepared”				24 h Soaked in 1.0 × 10^−4^ M H_2_O_2_			
Te(0) Binding Energy (eV)	Te(II) Binding Energy (eV)	Te(IV) Binding Energy (eV)	Te(IV)/Te(II) Ratio	Te(0) Binding Energy (eV)	Te(II) Binding Energy (eV)	Te(IV) Binding Energy (eV)	Te(IV)/Te(II) Ratio
571.7 ± 0.8	573.8 ± 0.5	575.9 ± 0.5	0.5 ± 0.1	571.3 ± 0.1	573.9 ± 0.1	576.3 ± 0.1	0.9 ± 0.2

**Table 4:** The position and ratios of Se (II) and Se (IV) peaks calculated for **TEOS-7** and **TEOS-8** xerogels “as prepared” and after submersion in 1.0 × 10^−4^ M (for **TEOS-7**) and 5.0 × 10^−5^ M (for **TEOS-8**) H_2_O_2_ in ASW for 24 h. The position and ratio values are the mean of five spectra recorded for each sample ± 1 SD. The peaks positions were referenced by setting a C 1s peak to 284.5 eV.

Xerogel	“as Prepared”			24 h Soaked in 1.0 × 10^−4^ or 5.0 × 10^−5^ M H_2_O_2_		
	Se(II) Binding Energy (eV)	Se(IV) Binding Energy (eV)	Se(II)/Se(IV) Ratio	Se(II) Binding Energy (eV)	Se(IV) Binding Energy (eV)	Se(IV)/Se(II) Ratio
**TEOS-7**	55.1 ± 0.2	--	--	55.4 ± 0.5	58.1 ± 0.5	0.2 ± 0.1
**TEOS-8**	56.1 ± 0.1	--	--	56.4 ± 0.2	58.8 ± 0.2	0.4 ± 0.2

**Figure 2:**
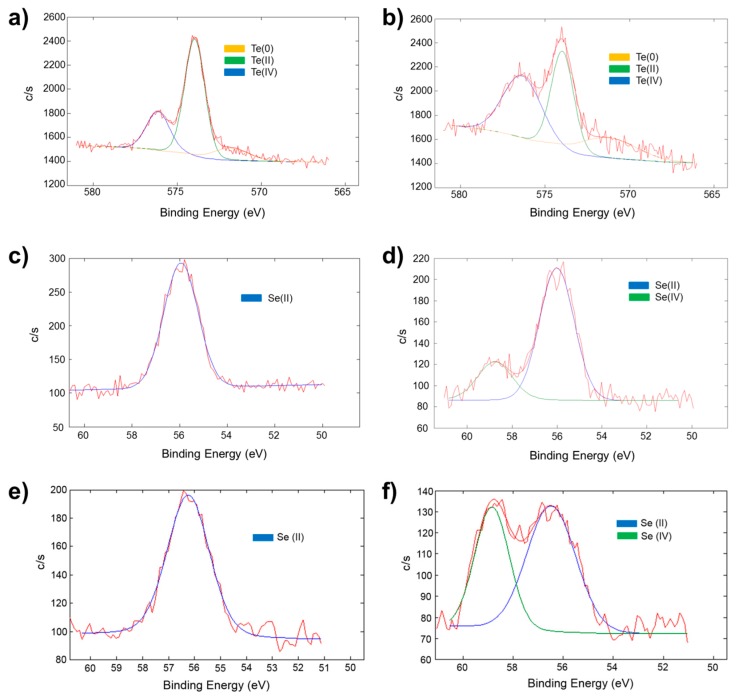
High resolution scans of (**a**) the Te 3d 5/2 peak region for the dry sample of **TEOS-3**; (**b**) the Te 3d 5/2 peak region for the **TEOS-3** sample soaked in 1.0 × 10^−4^ M H_2_O_2_ for 24 h; (**c**) the Se 3d peak region for the dry sample of **TEOS-7**; and (**d**) the Se 3d peak region for the **TEOS-7** sample soaked in 1.0 × 10^−4^ M H_2_O_2_ for 24 h; (**e**) the Se 3d peak region for the dry sample of **TEOS-8**; and (**f**) the Se 3d peak region for the **TEOS-8** sample soaked in 5.0 × 10^−5^ M H_2_O_2_ for 24 h.

The binding energies of the Te 3d 5/2 peaks for Te(II) (573.9 ± 0.1 eV) and Te(IV) (576.1 ± 0.1 eV) for the “as prepared” sample of **TEOS-3** correspond to previously reported values for the Te 3d 5/2 peaks in the Te(II) and Te(IV) oxidation state [34,35]. The position and ratios of the Te peaks were determined through deconvolution of a high resolution scan of Te 3d 5/2 peak region as seen in Figure 2a. The third peak corresponds to elemental Te, which is most likely generated and incorporated during the synthesis of the catalyst. The Te(IV) peak in Figure 2a is likely due to the oxidation of Te(II) by atmospheric oxygen, which would be expected to occur during the one-week drying time. Similar high resolution spectra were obtained for the **TEOS-7** catalyst by scanning the Se 3d region. Prior to exposure to H_2_O_2_, these catalysts showed only the Se(II) peak at 55.1 ± 0.1 eV during analysis of the “as prepared” sample (Figure 2c).

Upon exposure to 100 µM H_2_O_2_ the **TEOS-3** and **TEOS-7** catalysts are oxidized to the corresponding telluroxides and selenoxides as seen in Figure 2b,d, respectively. The peak positions and ratios of the chalogen(I1) and chalogen(IV) peaks are shown in Table 3 and Table 4. A significant growth in the Te(IV) peak (576.3 ± 0.1 eV) was observed after 24 h exposure to 1.0 × 10^−4^ M H_2_O_2_ in ASW (Student t-test, *p* < 0.05). After 24 h of soaking in 1.0 × 10^−4^ M H_2_O_2_ solution, the **TEOS-7** xerogel produced a second peak at 58.1 ± 0.1 eV corresponding to the Se(IV) oxidation state [36]. These data are consistent with oxidation of the telluride to the corresponding telluroxide in **TEOS-3** and oxidation of the selenide to selenoxide in **TEOS-7**.

The active catalyst in the **TEOS-8** xerogel is believed to be the seleninic acid, which reacts with H_2_O_2_ to form the perseleninic acid as the active oxidant [14,15]. Both seleninic and perseleninic acids have selenium in the +4 oxidation state and are formed by oxidation of diselenides in the +2 oxidation state [14,15]. To verify the production of Se(IV), 10 mol % of diselenide **8** was incorporated into a thin film composed of TEOS and analyzed using XPS. The **TEOS-8** film was analyzed prior to oxidation to verify only that Se(II) was in the film, then the film was soaked in 5.0 × 10^−5^ M H_2_O_2_ for 24 h, rinsed with deionized water and allowed to dry overnight exposed to air before a second analysis was performed. The high resolution scans of the Se 3d region are shown in Figure 2e,f before and after exposure to H_2_O_2_, respectively, and peak positions are recorded in Table 4. The data clearly indicate that only Se(II) is present in the film prior to oxidation (Se 3d at 56.1 eV) and is oxidized to Se(IV) upon exposure to H_2_O_2_ where a second peak at 58.8 ± 0.2 eV developed corresponding to the seleninic acid [37].

### 2.6. The Effect of Xerogel Composition on Rates of Reaction

**Figure 3:**
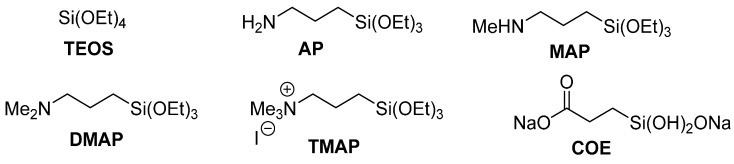
Structures of TEOS and the organically-modified siloxanes 3-aminopropyl- (triethoxy)silane (**AP**), 3-methylaminopropyl(triethoxy)silane (**MAP**), 3-dimethylamino-propyl(triethoxy)silane (**DMAP**), 3-triethoxysilylpropyltrimethylammonium iodide (**TMAP**), and carboxyethylsilanetriol, disodium salt (**COE**).

The rate of the reaction was probed using a series of xerogels monoliths with differing organic modifications. The organic groups on the monolith can alter the rate of the reaction through their interaction with the catalyst, through the formation of large pores, or by increasing the local concentration of reagents. The organosiloxanes utilized in this study in combination with TEOS are depicted in Figure 3.

Monoliths containing diselenide **8** were prepared from 10 mol % of an organosilane from Figure 3 (**AP**, **MAP**, **DMAP**, **TMAP**, or **COE**), 90 mol % TEOS, and 0.5 mol % **8** using aqueous HCl for hydrolysis. The sols were allowed to gel over 10 days at ambient conditions and the removal of solvents was accomplished after 48 h under vacuum. The monoliths were crushed to a fine white powder with mortar and pestle.

The ability of **8** sequestered in the various xerogel monoliths to catalyze the oxidation of bromide with H_2_O_2_ was again evaluated via the bromination of 4-pentenoic acid (**19**). The catalyst incorporated into a TEOS-only monolith and a catalyst-free TEOS monolith were used as controls. Results are compiled in Table 5. The reaction rate of the **8**-**TEOS** monolith was ~14× faster than the TEOS monolith without catalyst. The **AP**, **MAP**, and **DMAP**-containing xerogels gave modest rate accelerations (22%–65%) relative to the **8**-**TEOS** monolith as did the carboxylate-containing **COE** derivative (23%). In contrast, the incorporation of **TMAP** into the xerogel gave a >300% increase in the rate of catalysis relative to the **8**-**TEOS** monolith. Quaternary ammonium salts have been shown to generate large pores in silica [38], which might allow easier access for the reactants to enter the gel. Alternatively and perhaps more likely, the positive charge on the ammonium group could help concentrate/direct the negatively charged bromide ions giving a significant increase in rate due to an increase in the local concentration of bromide.

**Table 5:** Observed pseudo first-order rate constants (*k*_obs_) for bromination of 4-pentenoic acid (**19**) and relative rates (*k*_rel_) of bromination of 4-pentenoic acid (**19**) with catalyst **8** incorporated into varying xerogel formulations in pH 6.2 phosphate buffer ^a^.

Xerogel	*k*_obs_ (s^−1^) ^b^	*k*_rel_
Catalyst-free TEOS	(9.22 ± 0.04) × 10^−7^	0.07
TEOS	(1.24 ± 0.01) × 10^−5^	1.00
10:90 DMAP/TEOS	(1.61 ± 0.02) × 10^−5^	1.30
10:90 MAP/TEOS	(1.51 ± 0.03) × 10^−5^	1.22
10:90 AP/TEOS	(2.05 ± 0.06) × 10^−5^	1.65
10:90 TMAP/TEOS	(3.87 ± 0.01) × 10^−5^	3.12
10:90 COE/TEOS	(1.53 ± 0.04) × 10^−5^	1.23

^a^ Catalyst (0.025 equiv relative to substrate), 4-pentenoic acid (**19**, 0.138 M), NaBr (2.14 M), H_2_O_2_ (0.214 M); ^b^ *k*_obs_ is average of duplicate runs, T = 25 ± 1 °C.

## 3. Experimental Section

### 3.1. General Information

Tetraethoxysilane (TEOS), 3-aminopropyltriethoxysilane (AP), *N*-methylaminopropyltrimethoxysilane (MAP), 3-(*N*,*N*-dimethylaminopropyl)trimethoxysilane (DMAP), and carboxyethylsilanetriol, disodium salt (COE) were purchased from Gelest Inc. (Morrisville, PA, USA), The ditelluride compounds **9**, and **11**–**13** were prepared by literature procedures [18,39] Diphenyl diselenide (**10**) was purchased from Sigma-Aldrich (St. Louis, MO, USA). NMR spectra were recorded on an Inova 500 instrument (500 MHz for ^1^H, 125 MHz for ^13^C, Varian, Inc., Santa Clara, CA, USA) with residual solvent signal as internal standard. Infrared spectra were recorded on a Perkin-Elmer FTIR instrument (Perkin Elmer, Waltham, MA, USA). Mass spectra were acquired on a ThermoFinnigan MAT95XL high resolution magnetic sector mass spectrometer (Thermo Fisher Scientific, Waltham, MA, USA).

### 3.2. General Procedure for Kinetic Experiments

A stock solution of 0.23 M phosphate buffer (1:3.6 K_2_HPO_4_/KH_2_PO_4_ in 200 mL of D_2_O) containing 4-pentenoic acid (0.15 M) and propionic acid (0.01 M) was prepared with a final pH of 6.2. The catalyst (0.002 equiv based on total telluride relative to substrate for telluride series or 0.0025 equiv based on total selenide relative to substrate for selenide and diselenide series) and NaBr (7.5 mmol, 1.38 M for telluride, selenide, and diselenide series for kinetic studies and 11.25 mmol, 2.14 M for diselenide series recycling studies) were added to a centrifuge tube. The prepared stock solution was added (5 mL) followed by H_2_O_2_ (1.875 mmol, 0.346 M for telluride, selenide, and diselenide series for kinetic studies and 1.125 mmol, 0.214 M for diselenide series recycling studies). The reaction was timed beginning with the addition of H_2_O_2_. The mixture was allowed to stir at 25 ± 1 °C capped for the duration of the experiment. The experiment was run in duplicate and the calculated values of *k*_obs_ were reported as an average of the two trials. Throughout the experiment, the mixture was centrifuged and a small aliquot temporarily removed. The sample was analyzed by ^1^H-NMR spectroscopy: an internal alkene proton from 4-pentenoic acid was tracked alongside the methylene protons of the internal standard propionic acid. The experiment continued until the depletion of 4-pentenoic acid reached 75% consumption.

### 3.3. General Procedure for Recycling Catalysts

The initial reaction was run as described above until the consumption of 4-pentenoic acid was complete. The aqueous medium was removed via a Pasteur pipette following centrifugation of the reaction mixture. The remaining catalyst was washed with deionized water (3 × 10 mL). The catalyst was then washed with Et_2_O (3 × 10 mL) and the liquid removed. The washed catalyst was allowed to stand open to air for at least 18 h to allow excess solvent to evaporate. Once dry, the catalyst was recharged with same amount of NaBr, stock solution of buffer, pentenoic acid and propionic acid and H_2_O_2_ as previously described to begin the recycled kinetic experiment.

### 3.4. Preparation of Catalysts

The trialkoxysilane-containing compounds **1**–**8** all experienced hydrolysis of the alkoxysilyl functionality and oligomerization during attempts to isolate and characterize the materials. The oligomerization resulted in insoluble white solids. Compounds **3**, **7** and **8** were isolated by passing the reaction mixture through a short silica plug, concentrating and characterized by mass spectrometry and ^1^H-NMR spectroscopy, however the products continued to undergo hydrolysis and oligomerization. In practice, compounds **1**–**8** were immediately incorporated into the sol without isolation and characterization.

#### 3.4.1. Preparation of Trimethoxy(4-(phenyltelluranyl)phenyl)silane (**1**)

*p*-(Chloromethyl)phenyl(trimethoxy)silane (0.247 g, 1.00 mmol), diphenyl ditelluride (**9**, 0.102 g, 0.250 mmol) and NaBH_4_ (0.057 g, 1.50 mmol) were stirred under argon in anhydrous THF (2 mL). Methanol was added dropwise until the ditelluride was fully reduced (reaction turned colorless after <0.5 mL of MeOH) and the resulting mixture was then stirred overnight at ambient temperature. Anhydrous methanol (2 mL) was added to react with excess NaBH_4_ at the conclusion of reaction. The telluride **1**, as generated *in situ*, was used directly in the formation of the xerogel monoliths. All attempts to isolate **1** gave polymerized **1** as a white solid, which was insoluble in water, DMSO, and standard organic solvents.

#### 3.4.2. Preparation of Trimethoxy(4-(phenylselanyl)phenyl)silane (**2**)

*p*-(Chloromethyl)phenyl(trimethoxy)silane (70.0 mg, 0.300 mmol), diphenyl diselenide (**10**, 30.0 mg, 0.100 mmol) and NaBH_4_ (20.0 mg, 0.600 mmol) were treated as described for the preparation of **1** except that EtOH was substituted for MeOH. The product, as generated *in situ*, was used directly in the formation of the xerogel monoliths. For characterization, following an aqueous workup and extraction with CH_2_Cl_2_, crude selenide **2** was isolated as a yellow oil in 65% yield: ^1^H-NMR (CDCl_3_, 300 MHz): δ 7.53 (m, 2 H), 7.44 (m, 2 H), 7.22 (m, 5 H), 3.61 (s, <9 H), 3.57 (s, 2 H); HRMS (ESI), *m/z* 368.0357 (calcd for [C_16_H_20_O_3_Si^80^Se]^+^ 368.0341).

#### 3.4.3. Preparation of (3-(Phenyltelluranyl)propyl)silane (**3**)

3-Chloropropyltriethoxysilane (0.241 g, 1.00 mmol), diphenyl ditelluride (**9**, 0.102 g, 0.250 mmol) and NaBH_4_ (0.057 g, 1.50 mmol) were treated as described for the preparation of **1**. The product, as generated *in situ*, was used directly in the formation of the xerogel monoliths. Initial attempts to isolate **3** following an aqueous workup gave a “reactive” oil, which degraded even under an inert atmosphere presumably due to hydrolysis of the ethoxysilane functionality. For characterization, following direct isolation from a silica plug eluted with hexanes/CH_2_Cl_2_, crude telluride **3** was isolated as an orange oil in 39% isolated yield: ^1^H-NMR (CDCl_3_, 500 MHz): δ 7.70 (m, 2 H), 7.25 (m, 1 H), 7.18 (m, 2 H), 3.78 (q, *J* = 6.9 Hz, 6 H), 2.94 (t, *J* = 7.5 Hz, 2 H), 1.90 (quint, *J* = 8.1 Hz, 2 H), 1.20 (t, *J* = 6.9 Hz, 9 H), 0.74 (t, *J* = 8.1 Hz, 2 H); HRMS (ESI), *m*/*z* 435.0606 (calcd for [C_15_H_26_O_3_Si^130^Te+Na]^+^ 435.0609).

#### 3.4.4. Preparation of 4-((3-(Triethoxysilyl)propyl)telluranyl)aniline (**4**)

3-Chloropropyltriethoxysilane (0.241 g, 1.00 mmol), bis(4-(*N*,*N*-dimethylaniline))ditelluride (**11**, 0.124 g, 0.250 mmol) and NaBH_4_ (0.057 g, 1.50 mmol) were treated as described for the preparation of **1**. The product, as generated *in situ*, was used directly in the formation of the xerogel monoliths. All attempts to isolate **4** gave polymerized **4** as a white solid, which was insoluble in water, DMSO, and standard organic solvents.

#### 3.4.5. Preparation of (3-((4-Chlorophenyl)telluranyl)propyl)triethoxysilane (**5**)

3-Chloropropyltriethoxysilane (0.241 g, 1.00 mmol), bis(4-chlorophenyl)ditelluride (**12**, 0.120 g, 0.250 mmol) and NaBH_4_ (0.057 g, 1.50 mmol) were treated as described for the preparation of **1**. The product, as generated *in situ*, was used directly in the formation of the xerogel monoliths. All attempts to isolate **5** gave polymerized **5** as a white solid, which was insoluble in water, DMSO, and standard organic solvents.

#### 3.4.6. Preparation of Triethoxy(3-((4-methoxyphenyl)telluranyl)propyl)silane (**6**)

3-Chloropropyltriethoxysilane (0.241 g, 1.00 mmol), bis(4-methoxyphenyl)ditelluride (**13**, 0.117 g, 0.250 mmol) and NaBH_4_ (0.057 g, 1.50 mmol) were treated as described for the preparation of **1**. The product, as generated *in situ*, was used directly in the formation of the xerogel monoliths. All attempts to isolate **6** gave polymerized **6** as a white solid, which was insoluble in water, DMSO, and standard organic solvents.

#### 3.4.7. Preparation of Triethoxy(3-(phenylselanyl)propyl)silane (**7**)

3-Chloropropyltriethoxysilane (0.380 g, 1.50 mmol), diphenyl diselenide (**10**, 0.160 g, 0.500 mmol) and NaBH_4_ (60.0 mg, 1.50 mmol) were treated as described for the preparation of **1** except that EtOH was substituted for MeOH. The product, as generated *in situ*, was used directly in the formation of the xerogel monoliths due to rapid hydrolysis of the triethoxysilyl ethoxy groups. Initial characterization of **7**, isolated as a yellow oil following direct isolation from a silica plug eluted with hexanes/EtOAc, gave crude **7** in 91% yield: ^1^H-NMR (CDCl_3_, 500 MHz): δ 7.48 (d, *J* = 7 Hz, 2 H), 7.23 (m, 3 H), 3.78 (q, *J* = 7.5 Hz, <6 H), 2.95 (t, *J* = 7.5 Hz, 2 H), 1.81 (quint, *J* = 8.5 Hz, 2 H), 1.20 (t, *J* = 7.5 Hz, <9 H), 0.77 (t, *J* = 8 Hz, 2 H). HRMS (ESI), *m*/*z* 385.0709 (calcd for [C_15_H_26_O_3_Si^80^Se+Na]^+^ 385.0714).

#### 3.4.8. Synthesis of 2,2′-Diselanediyldibenzoic Acid (**16**)

Ethyl-2-selenocyanatobenzoate (**15**) was prepared following literature procedures [14]. A LiOH solution (0.300 M, 140 mL) was added into a stirring solution of **15** (2.12 g, 8.35 mmol) dissolved in THF (100 mL) and allowed to stir at room temperature for 6 h open to air. 1 M HCl (90.0 mL, 90.0 mmol) was added until the solution reaches pH ≈ 2. The solution was extracted with EtOAc (3 × 50 mL), the combined organic layers dried over MgSO_4_ and concentrated. The product was purified through recrystallization using EtOAc/hexanes to give a white solid. Spectral data agreed with published spectra: [40] m.p. 180–183 °C, yield 40%, ^1^H-NMR (CD_3_OD, 500 MHz): δ 8.07 (d, *J* = 7.5 Hz, 2 H), 7.92 (d, *J* = 8.1 Hz, 2 H), 7.58 (t, *J* = 7.8 Hz, 2 H), 7.41 (t, *J* = 7.2 Hz, 2 H). HRMS (ESI), *m*/*z* 424.9 (calcd for [C_14_H_10_NaO_4_Se_2_]^+^ 424.9).

#### 3.4.9. Synthesis of 2,2′-Diselanedibenzoyl Chloride [41] (**17**)

Thionyl chloride (0.100 mL, 1.35 mmol) was added to a flame dried flask fitted with a reflux condenser containing a refluxing solution of o-carboxylic acid diselenide (0.130 g, 0.340 mmol) in benzene (5 mL). The solution was refluxed for 3 h then allowed to cool to ambient temperature. The solution was concentrated to give a yellow solid in 97% yield and the crude product used in the next step without further purification: m.p. 118–120 °C, ^1^H-NMR (CDCl_3_, 500 MHz): δ 8.45 (d, *J* = 8.1 Hz, 2 H), 8.14 (d, *J* = 8.1 Hz, 2 H), 7.75 (t, *J* = 8.4 Hz, 2 H), 7.58 (t, *J* = 8.4 Hz, 2 H).

#### 3.4.10. Synthesis of 2,2′-Diselanediylbis(*N*-(3-(triethoxysilyl)propyl)benzamide **8**

3-Aminopropyltriethoxysilane (0.153 mL, 0.650 mmol) was added into a stirring solution of acid chloride (0.140 g, 0.330 mmol) dissolved in dry CH_2_Cl_2_ (6 mL). The solution was allowed to stir for 12 h under argon. The resulting solution was concentrated to give a yellow oil and used immediately in the next step without further purification due to hydrolysis of the ethoxysilane functionality. For the crude product: ^1^H-NMR (CDCl_3_, 500 MHz): δ 8.12 (d, *J* = 8 Hz, 2 H), 7.68 (d, *J* = 7.5 Hz, 2 H), 7.54 (t, *J* = 7.5 Hz, 2 H), 7.41 (t, *J* = 7 Hz, 2 H), 3.49 (q, *J* = 6.5, <12 H), 3.01 (t, *J* = 1.89 (quint, *J* = 7.5 Hz, 4 H), 1.23 (t, *J* = 7 Hz, <18 H), 0.72 (t, *J* = 7 Hz, 4 H). HRMS (ESI), *m*/*z* 809.1666 (calcd for [C_32_H_53_N_2_O_8_Si_2_^80^Se_2_]^+^ 809.1667).

### 3.5. Preparation of Monoliths

#### 3.5.1. Preparation of 2.5 mol % **1** in TEOS

TEOS (4.46 mL, 20.0 mmol) was added to a stirring solution of **1** (0.500 mmol) in EtOH (4.66 mL, 80.0 mmol). A solution of 14.5 M NH_4_OH (0.138 mL, 2.00 mmol) and deionized water (3.24 mL, 180 mmol) was added to the reaction slowly. The resulting solution was allowed to stir uncapped overnight, then remained open to air for 10 days until a dense gel formed. The gel was placed in a vacuum oven at ambient temperature for 48 h, then crushed into a fine powder using a mortar and pestle. The resulting monolith was washed with ethyl ether (3 × 10 mL) to check for leaching (by ^1^H-NMR spectroscopy and MS). The monolith was dried for an additional 2 h in a vacuum oven at ambient temperature before use.

#### 3.5.2. Preparation of 0.5 mol % **2** in TEOS

TEOS (8.93 mL, 40.0 mmol) was added to a stirring solution of **2** (0.200 mmol) in EtOH (9.33 mL, 160 mmol). A solution of 14.5 M NH_4_OH (0.280 mL, 4.06 mmol) and deionized water (5.76 mL, 320 mmol) was added to the reaction slowly and the resulting solution allowed to gel. The resulting monolith was treated as previously described.

#### 3.5.3. Preparation of 2.5 mol % **3** in TEOS

TEOS (4.46 mL, 20.0 mmol) was added to a stirring solution of **3** (0.500 mmol) in EtOH (4.66 mL, 80.0 mmol). A solution of 14.5 M NH_4_OH (0.138 mL, 2.00 mmol) and deionized water (3.24 mL, 180 mmol) was added to the reaction slowly and the resulting solution allowed to gel. The resulting monolith was treated as previously described.

#### 3.5.4. Preparation of 2.5 mol % **4** in TEOS

TEOS (4.63 mL, 20.0 mmol) was added to a stirring solution of **4** (0.500 mmol) in EtOH (4.66 mL, 80.0 mmol). A solution of 14.5 M NH_4_OH (0.138 mL, 2.00 mmol) and deionized water (3.24 mL, 180 mmol) was added to the reaction slowly and the resulting solution allowed to gel. The resulting monolith was treated as previously described.

#### 3.5.5. Preparation of 2.5 mol % **5** in TEOS

TEOS (4.46 mL, 20.0 mmol) was added to a stirring solution of **5** (0.500 mmol) in EtOH (4.66 mL, 80.0 mmol). A solution of 14.5 M NH_4_OH (0.138 mL, 2.00 mmol) and deionized water (3.24 mL, 180 mmol) was added to the reaction slowly and the resulting solution allowed to gel. The resulting monolith was treated as previously described.

#### 3.5.6. Preparation of 2.5 mol % **6** in TEOS

TEOS (4.46 mL, 20.0 mmol) was added to a stirring solution of **5** (0.500 mmol) in EtOH (4.66 mL, 80.0 mmol). A solution of 14.5 M NH_4_OH (0.138 mL, 2.00 mmol) and deionized water (3.24 mL, 180 mmol) was added to the reaction slowly and the resulting solution allowed to gel. The resulting monolith was treated as previously described.

#### 3.5.7. Preparation of 0.5 mol % **7** in TEOS

TEOS (8.93 mL, 40.0 mmol) was added to a stirring solution of **7** (0.200 mmol) in EtOH (9.33 mL, 160 mmol). A solution of 14.5 M NH_4_OH (0.280 mL, 4.06 mmol) and deionized water (5.76 mL, 320 mmol) was added to the reaction slowly and the resulting solution allowed to gel. The resulting monolith was treated as previously described.

#### 3.5.8. Preparation of 0.5 mol % **8** in TEOS

TEOS (10.6 mL, 47.4 mmol) was added to a stirring solution of **8** (0.240 mmol) in EtOH (11.1 mL, 190 mmol). A solution of 12 M HCl (0.790 mL, 9.48 mmol) and deionized water (6.83 mL, 379 mmol) was added to the reaction slowly and the resulting solution allowed to gel. The resulting monolith was treated as previously described.

#### 3.5.9. Preparation of 0.5 mol % **8** in 10:90 DMAP/TEOS

TEOS (7.27 mL, 32.6 mmol) and DMAP (0.792 mL, 3.62 mmol) was added to a stirring solution of **8** (0.181 mmol) in EtOH (7.60 mL, 130 mmol). A solution of 12 M HCl (0.543 mL, 6.52 mmol) and deionized water (4.69 mL, 261 mmol) was added to the reaction slowly and the resulting solution allowed to gel. The resulting monolith was treated as previously described.

#### 3.5.10. Preparation of 0.5 mol % **8** in 10:90 MAP/TEOS

TEOS (7.27 mL, 32.6 mmol) and MAP (0.716 mL, 3.62 mmol) was added to a stirring solution of **8** (0.181 mmol) in EtOH (7.60 mL, 130 mmol). A solution of 12 M HCl (0.543 mL, 6.52 mmol) and deionized water (4.69 mL, 261 mmol) was added to the reaction slowly and the resulting solution allowed to gel. The resulting monolith was treated as previously described.

#### 3.5.11. Preparation of 0.5 mol % **8** in 10:90 AP/TEOS

TEOS (7.27 mL, 32.6 mmol) and AP (0.851 mL, 3.62 mmol) was added to a stirring solution of **8** (0.181 mmol) in EtOH (7.60 mL, 130 mmol). A solution of 12 M HCl (0.543 mL, 6.52 mmol) and deionized water (4.69 mL, 261 mmol) was added to the reaction slowly and the resulting solution allowed to gel. The resulting monolith was treated as previously described.

#### 3.5.12. Preparation of 0.5 mol % **8** in 10:90 COE/TEOS

First, a sol COE was prepared by adding COE (1.73 g, 2.20 mmol) and EtOH (5.38 mL) into a vial. A solution of 12 M HCl (0.376 mL, 4.51 mmol) and deionized water (0.198 mL, 11.0 mmol) was added to the vial and the resulting mixture allowed to stir for 1 h. A sol TEOS solution was prepared by dissolving **8** (0.11 mmol) in EtOH (4.62 mL, 79.2 mmol) and adding TEOS (4.42 mL, 19.8 mmol) and a solution of 12 M HCl (0.112 mL, 1.33 mmol) and deionized water (2.85 mL, 158 mmol). The solution was stirred uncapped for 1 h at ambient temperature. The sol COE was then added to the sol TEOS by passing it through a cotton plug to remove the salts. The solution was allowed to gel. The resulting monolith was treated as previously described.

#### 3.5.13. Synthesis of 3-Trimethoxysilyl-Propyltrimethylammonium Iodide [[19]] (TMAP)

Iodomethane (4.21 mL, 67.8 mmol) was added to a refluxing mixture of KHCO_3_ (7.49 g, 54.2 mmol), AP (3.18 mL, 13.6 mmol) and acetone (15 mL). The resulting mixture was allowed to reflux overnight, then cooled to ambient temperature and the salts filtered. The filtrate was concentrated, dissolved in CH_2_Cl_2_ and the KI removed by filtration. The pure product was isolated after recrystallization in CH_2_Cl_2_/diethyl ether as a while solid (yield 70%). ^1^H-NMR (D_2_O, 500 MHz) δ 3.82 (q, *J* = 6.5 Hz, 6 H), 3.24 (t, *J* = 8.5 Hz, 2 H), 3.02 (s, 9 H), 1.73 (quint, *J* = 3.5 Hz, 2 H), 1.11 (t, *J* = 7 Hz, 9 H), 0.64 (t, *J* = 9 Hz, 2 H); LRMS (ESI), *m*/*z* 264.2 (calcd for [C_12_H_30_NO_3_Si]^+^ 264.1)

#### 3.5.14. Preparation of 0.5 mol % **8** in 10:90 TMAP/TEOS

TEOS (4.58 mL, 20.5 mmol) and TMAP (0.892 g, 2.28 mmol) were added to a stirring solution of **8** (0.114 mmol) in EtOH (4.81 mL, 82.5 mmol). A solution of 12 M HCl (0.344 mL, 4.12 mmol) and deionized water (2.97 mL, 165 mmol) was added to the reaction slowly and the resulting solution allowed to gel. The resulting monolith was treated as previously described.

The iodide counter ion was exchanged for a bromide counter ion by adding NaBr (0.830 M), 4-pentenoic acid (0.280 M) to a vial containing the monolith followed by the pH 6.1 buffer solution (15 mL) and H_2_O_2_ (0.420 mL). After 30 min the monolith turned from dark orange to white. The reaction was allowed to stir for 5 h, then exacted with Et_2_O (3 × 15 mL). The monolith was then rinsed with deionized water (3 × 15 mL) and after centrifugation the aqueous medium was removed from the monolith. The monolith was allowed to dry open to air, then dried in the vacuum oven for 24 h at ambient temperature to ensure the water had been removed.

### 3.6. Formation of Thin Films

#### 3.6.1. Cleaning Microscope Slides

Glass microscope slides (25 mm × 75 mm), purchased from Fisher Scientific (Waltham, MA, USA), were submerged in freshly prepared aqueous Piranha solution composed of 1:4 30% H_2_O_2_/sulfuric acid. After 24 h the slides were rinsed with copious amounts of deionized water and placed in isopropanol for at least 15 min before coating.

#### 3.6.2. Spin Coating

A Spincoater^®^ Model P6700 Series (Specialty Coating Systems, Inc., Indianapolis, IN, USA) was used to form the xerogel coating on the slide. Using a micropipette, 400 μL of sol was applied to the slide as it spun for 10 s at 100 revolutions per minute (RPM). The sol was then cast onto the slide by spinning at 3000 RPM for 60 s. The resulting coating was stored at ambient temperature for one week before analysis.

#### 3.6.3. Preparation of 5 mol % **3** in TEOS

TEOS (4.46 mL, 20.0 mmol) was added to a stirring solution of **3** (1.00 mmol) in EtOH (4.67 mL). A solution of HCl (12 M, 0.10 mL) and deionized water (2.88 mL) were added slowly and the resulting solution was stirred over night at ambient temperature before coating.

#### 3.6.4. Preparation of 20 mol % **7** in TEOS

TEOS (4.46 mL, 20.0 mmol) was added to a stirring solution of **7** (4.00 mmol) in EtOH (4.67 mL, 80.0 mmol). A solution of 12 M HCl (0.100 mL, 1.20 mmol) and deionized water (2.88 mL, 160 mmol) were added slowly and the resulting solution was stirred over night at ambient temperature before coating.

#### 3.6.5. Preparation of 10 mol % (**8**) in TEOS

TEOS (0.513 mL, 2.30 mmol) was added to a stirring solution of **8** (0.230 mmol) in EtOH (0.531 mL, 9.11 mmol). A solution of 12 M HCl (11.0 µL, 0.127 mmol) and deionized water (0.328 mL, 18.2 mmol) were added slowly and the resulting solution was stirred over night at ambient temperature before coating.

### 3.7. X-ray Photoelectron Spectroscopy

The elemental composition and chemical state for each of the coating surfaces were examined by X-ray photoelectron spectroscopy (XPS) for both before and after immersion in ASW. The slides were cut into duplicate samples using a glass cutter. The samples were analyzed using a Model 500 VersaProbe equipped with an Al source, a hemispherical analyzer and a sixteen channel detector (Physical Electronics Laboratories (PHI), Chanhassen, MN, USA). A takeoff angle of 45° was used to obtain spectra. A monochromatic Al kα source (1486.6 eV) was operated at 100 µm 25 W 15 kV. The operating pressure in the main chamber did not exceed 5 × 10^−6^ Pa. A pass energy of 117.4 eV was used to obtain a survey scan and a pass energy of 23.5 eV was used for high resolution scans. Curve fitting was performed using PHI MultiPak™ Software version 8 (PHI).

Samples were initially analyzed after storage at ambient temperatures for at least 1 week. The same sample was then oxidized with H_2_O_2_ by soaking the samples in ASW containing either 50 or 100 µM H_2_O_2_ for 24 h. Samples were then allowed to air-dry overnight to allow the bulk of the water to evaporate from the surface before analyzing the sample again.

The background levels of carbon (C1s) and nitrogen (N1s) were measured using clean glass slides [20]. The detected level of C(1s)/Si(2p3) was 0.2 and the levels of N(1s)/Si(2p3). All of the recorded ratios for the xerogel coatings were significantly larger (*p* ˃ 0.05) than the background levels as determined by pairwise comparison.

## 4. Conclusions

Tellurides, selenoxides, and seleninic acids all function as catalysts for the activation of hydrogen peroxide. For each of these catalyst classes, the chalcogen atom is the center of interaction with hydrogen peroxide. Here, we have examined these catalyst centers sequestered in xerogel formulations to probe the sensitivity of the catalyst centers to the local environment, the “robustness” of the catalyst-xerogel combinations to recovery and reuse—a necessity for any long-term synthetic viability—as well as potential lifetime as an antifouling surface in a marine environment.

The benzyl-substituted systems **TEOS-1** and **TEOS-2**, while highly active as catalysts, have limited viability since most catalytic activity is lost when the xerogel is recovered and reused. This suggests that the chalcogen atoms do not remain sequestered in the xerogel, but likely produce degradation products that are active catalysts in solution. Recovery removes the soluble component from further reaction.

The **TEOS**-**3** through **TEOS-6** combinations all show catalytic activity and the rate of bromination with **TEOS-3** remains unchanged after four cycles of reaction (2000 turnovers). The selenoxide analogue of **TEOS-3**—the **TEOS-7** combination oxidized to the selenoxide **TEOS-24**—is not as robust and likely undergoes a *syn*-elimination of PhSeOH, which again becomes a soluble catalyst (perhaps oxidized to PhSeO_2_H) that is lost upon xerogel recovery.

The **TEOS-8** combination is presumably oxidized to the seleninic acid-containing **TEOS-25**, which is a robust catalyst with catalytic rates unchanged after four cycles. The incorporation of **8** into organically-modified xerogels can give enhanced rates with 10 mol % **TMAP**/**TEOS** combination giving a >300% increase in rate. The positive charge on **TMAP** may help concentrate bromide ions to facilitate reaction.

Surface analysis of each of the telluride, selenide, and diselenide catalysts/catalyst precursors by XPS shows an increase in the chalcogen(IV) oxidation state upon exposure to H_2_O_2_. These results are consistent with the speculated mechanisms involving telluroxide/(dihydroxy)tellurane intermediates (Te(IV)), selenoxides and hydroxyl(perhydroxy)selenane intermediates (Se(IV)), and seleninic acid/perseleninic acid intermediates (Se(IV)].

The data presented here can be incorporated into next-generation xerogel-sequestered systems with improved catalytic activity and lifetime. These catalysts will likely be based on telluride or seleninic acid functionality sequestered in xerogels tailored to direct the negatively charged halide ions to the active site.

## References

[1] Strukul G. (1992). Catalytic Oxidations with Hydrogen Peroxide as Oxidant.

[2] Ten Brink G.J., Vis J.M., Arends I.W.C.E., Sheldon R.A. (2001). Selenium-Catalyzed Oxidations with Aqueous Hydrogen Peroxide. 2. Baeyer-Villiger Reactions in Homogeneous Solution. J. Org. Chem..

[3] Ten Brink G.J., Fernandes B.C.M., van Vliet M.C.A., Arends I.W.C.E., Sheldon R.A. (2001). Selenium catalyzed oxidations with aqueous hydrogen peroxide. Part I. Epoxidation reactions in homogenous solution. J. Chem. Soc. Perkin Trans. 1.

[4] Drabowicz J., Mikolajczyk M. (1978). A Facile and Selective Oxidation of Organic Sulphides and Sulphoxides with Hydrogen Peroxide/Selenium Dioxide System. Synthesis.

[5] Reich H.J., Chow F., Peake S.L. (1978). Seleninic Acids as Catalysts for Oxidation of Olefins and Sulfides Using Hydrogen Peroxide. Synthesis.

[6] Ten Brink G.J., Vis J.M., Arends I.W.C.E., Sheldon R.A. (2002). Selenium catalysed oxidations with aqueous hydrogen peroxide. Part 3. Oxidation of carbonyl compounds under mono/bi/triphasic conditions. Tetrahedron.

[7] Murahashi S., Tatsuki S. (1987). Selenium Dioxide Catalyzed Oxidation of Secondary Amines with Hydrogen Peroxide. Simple Synthesis of Nitrones from Secondary Amines. Tetrahedron Lett..

[8] Back T.G., Moussa Z. (2002). Remarkable activity of a novel phenylseleninate ester as a glutathione peroxidase mimetic and its facile *in situ* generation from allyl 3-hydroxypropyl selenide. J. Am. Chem. Soc..

[9] Press D.J., McNeil N.M.R., Hambrook M., Back T.G. (2014). Effects of methoxy substituents on glutathione peroxidase-like activity of cyclic seleninate esters. J. Org. Chem..

[10] Mugesh G., Singh H.B. (2000). Synthetic organoselenium compounds as antioxidants: Glutathione peroxidaze activity. Chem. Soc. Rev..

[11] Goodman M.A., Detty M.R. (2004). Selenoxides as Catalysts for the Activation of Hydrogen Peroxide. Bromination of Organic Substrates with Sodium Bromide and Hydrogen Peroxide. Organometallics.

[12] Francavilla C., Drake M.D., Bright F.V., Detty M.R. (2001). Dendrimeric Organochalcogen Catalysts for the Activation of Hydrogen Peroxide: Improved Catalyst Activity through Statistical Effects and Cooperativity in Successive Generations. J. Am. Chem. Soc..

[13] Drake M.D., Bright F.V., Detty M.R. (2003). Dendrimeric Organochalcogen Catalyst for the Activation of Hydrogen Peroxide: Origins of the “Dendrimer Effect” with Catalyst Terminating in Phenylseleno Groups. J. Am. Chem. Soc..

[14] Alberto E.E., Braga A.L., Detty M.R. (2012). Imidazolium-containing diselenides for catalytic oxidations with hydrogen peroxide and sodium bromide in aqueous solutions. Tetrahedron.

[15] Drake M.D., Bateman M.A., Detty M.R. (2003). Substituent Effects in Arylseleninic Acid-Catalyzed Bromination of Organic Substrates with Sodium Bromide and Hydrogen Peroxide. Organometallics.

[16] Detty M.R., Zhou F., Friedman A.E. (1996). Positive Halogens from Halides and Hydrogen Peroxide with Organotellurium Catalysts. J. Am. Chem. Soc..

[17] Higgs D.E., Nelen M.I., Detty M.R. (2001). Iodination of Organic Substrates with Halide Salts and H_2_O_2_ Using an Organotelluride Catalyst. Org. Lett..

[18] Alberto E.E., Muller L.M., Detty M.R. (2014). Rate Accelerations of Bromination Reactions with NaBr and H_2_O_2_ via the Addition of Catalytic Quantities of Diaryl Ditellurides. Organometallics.

[19] Bennett S.M., Ying T., McMaster D., Bright F.V., Detty M.R. (2008). A Xerogel-Sequestered Selenoxide Catalyst for Bromination with Hydrogen Peroxide and Sodium Bromide in an Aqueous Environment. J. Org. Chem..

[20] Tang Y., Finlay J.A., Kowalke G.L., Meyer A.E., Bright F.V., Callow M.E., Callow J.A., Wendt D.E., Detty M.R. (2005). Hybrid xerogel films as novel coatings for antifouling and fouling release. Biofouling.

[21] Bennett S.M., Finlay J.A., Gunari N., Wells D.D., Meyer A.E., Walker G.C., Callow M.E., Callow J.A., Bright F.V., Detty M.R. (2010). The role of surface energy and water wettability in aminoalkyl/fluorocarbon/hydrocarbon-modified xerogel surfaces in the control of marine biofouling. Biofouling.

[22] Evariste E., Gatley C.M., Detty M.R., Callow M.E., Callow J.A. (2013). The performance of aminoalkyl/fluorocarbon/hydrocarbon-modified xerogel coatings against the marine alga Ectocarpus crouaniorum: Relative roles of surface energy and charge. Biofouling.

[23] Finlay J.A., Bennett S.M., Brewer L.H., Sokolova A., Clay G., Gunari N., Meyer A.E., Walker G.C., Wendt D.E., Callow M.E. (2010). Barnacle settlement and the adhesion of protein and diatom microfouling to xerogel films with varying surface energy and water wettability. Biofouling.

[24] Sokolova A., Bailey J.J., Waltz G.T., Brewer L.H., Finlay J.A., Fornalik J., Wendt D.E., Callow M.E., Callow J.A., Bright F.V. (2012). Spontaneous multiscale phase separation within flurinated xerogel coatings for fouling-release surfaces. Biofouling.

[25] Sokolova A., Cilz N., Daniels J., Stafslien S.J., Brewer L.H., Wendt D.E., Bright F.V., Detty M.R. (2012). A comparison of the antifouling/foul-release characteristics of non-biocidal xerogel and commerical coatings towards micro- and macrofouling organsims. Biofouling.

[26] Gunari N., Brewer L.H., Bennett S.M., Sokolova A., Kraut N.D., Finlay J.A., Meyer A.E., Walker G.C., Wendt D.E., Callow M.E. (2011). The control of marine biofouling on xerogel surfaces with nanometer-scale topography. Biofouling.

[27] Selvaggio P., Tusa S., Detty M.R., Bright F.V., Ciriminna R., Pagliaro M. (2009). Ecofriendly protection from biofouling of the monitoring system as Pantelleria’s Cala Gadir underwater archaeological site, Sicily. Int. J. Naut. Arch..

[28] McMaster D.M., Bennett S.M., Tang Y., Finlay J.A., Kowalke G.L., Nedved B., Bright F.V., Callow M.E., Callow J.A., Wendt D.E. (2009). Antifouling character of “active” hybrid xerogel coatings with sequestered catalysts for the activation of hydrogen peroxide. Biofouling.

[29] Reich H.J., Hoeger C.A., Willis W.W. (1982). Organoselenium Chemistry. Characterization of Reactive Intermediates in the Selenoxide Syn Elimination: Selenenic Acids and Selenolseleninate Esters. J. Am. Chem. Soc..

[30] Reich H.J., Reich I.L., Renga J.M. (1973). Organoselenium Chemstiry. α-Phenylseleno Carbonyl Compounds as Precursors for α,β-Unsaturated Ketones and Esters. J. Am. Chem. Soc..

[31] Reich H.J., Renga J.M., Reich I.L. (1975). Organoselenium Chemistry. Converstion of Ketones to Enones by Selenoxide Syn Elimination. J. Am. Chem. Soc..

[32] Sharpless K.B., Young M.W., Lauer R.F. (1973). Reactions of Selenoxides: Thermal Syn-elimination and H_2_^18^O Exchange. Tetrahedron Lett..

[33] Vickerman J.C., Gilmore I.S. (2009). Surface Analysis-The Principal Techniques.

[34] Detty M.R., Lenhart W.C., Gassman P.G., Callstrom M.R. (1989). XPS and ^125^Te NMR of Organotellurium Compounds. 2. Oxatellurolyium Halides and Dioxatellurapentalenes and Their Products of Oxidative Halogen Addition. Organometallics.

[35] Detty M.R., Lenhart W.C., Gassman P.G., Callstrom M.R. (1989). XPS and ^125^Te NMR studies of organotellurium compounds. I. Tellurapyrans, tellurapyranones, tellurapyrylium salts, and their benzo analogues in both the tellurium(II) and tellurium(IV) oxidation states. Organometallics.

[36] Shenasa M., Sainkar S., Lichtman D. (1986). XPS study of some selected selenium compounds. J. Electron. Spectrosc. Relat. Phenom..

[37] Wang L., Cao W., Yi Y., Xu H. (2014). Dual redox responsive coassemblies of diselenide-containing block copolymers and polymer lipids. Langmuir.

[38] Hoffmann F., Cornelius M., Morell J., Froba M. (2006). Silica-based mesoporous organic-inorganic hybrid materials. Angew. Chem..

[39] Engman L., Stern D., Cotgreave I.A., Andersson C.M. (1992). Thiol Peroxidase Activity of Diaryl Ditellurides as Determined by a ^1^H-NMR Method. J. Am. Chem. Soc..

[40] Erben F., Kleeblatt D., Sonneck M., Hein M., Feist H., Fahrenwaldt T., Fischer C., Matin A., Iqbal J., Plotz M. (2013). Synthesis and antiproliferative activity of selenoindirubins and selenoindirubin-*N*-glycosides. Org. Biomol. Chem..

[41] Lou Z., Li P., Sun X., Yang S., Wang B., Han K. (2013). A fluorescent probe for rapid detection of thiols and imaging of thiols reducing repair and H_2_O_2_ oxidative stress cycles in living cells. Chem. Commun. (Camb.).

